# Interleukin-6 induces fat loss in cancer cachexia by promoting white adipose tissue lipolysis and browning

**DOI:** 10.1186/s12944-018-0657-0

**Published:** 2018-01-16

**Authors:** Jun Han, Qingyang Meng, Lei Shen, Guohao Wu

**Affiliations:** Department of General Surgery, Zhongshan Hospital of Fudan University, 180 Fenglin Road, Shanghai, 200032 People’s Republic of China

**Keywords:** Interleukin-6, Cancer cachexia, Lipolysis, Beige adipocyte

## Abstract

**Background:**

Cancer cachexia is a progressive and multi-factorial metabolic syndrome characterized by loss of adipose tissue and skeletal muscle. White adipose tissue (WAT) lipolysis and white-to-brown transdifferentiation of WAT (WAT browning) are proposed to contribute to WAT atrophy in cancer cachexia. Chronic inflammation, mediated by cytokines such as tumor necrosis factor alpha (TNF-α) and interleukin-6 (IL-6), has been reported to promote cancer cachexia. However, whether chronic inflammation promotes cancer cachexia by regulating WAT metabolism and the underlying mechanism remains unclear.

**Methods:**

In this study, we first analyzed the association between chronic inflammation and WAT metabolism in gastric and colorectal cancer cachectic patients. In cachectic mice treated with anti-IL-6 receptor antibody, we clarified whether WAT lipolysis and browning were regulated by IL-6.

**Results:**

Clinical analyses showed positive significant association between serum IL-6 and free fatty acid (FFA) both in early- and late-stage cancer cachexia. However, serum TNF-α was positively associated with serum FFA in the early- but not late-stage cachexia. WAT lipolysis was increased in early- and late-stage cachexia, while WAT browning was detected only in late-stage cachexia. Anti-IL-6 receptor antibody inhibited WAT lipolysis and browning in cachectic mice.

**Conclusions:**

Based on these findings, we conclude that chronic inflammation (especially that mediated by IL-6) might promote cancer cachexia by regulating WAT lipolysis in early-stage cachexia and browning in late-stage cachexia.

## Background

Cancer cachexia is a wasting syndrome defined by an ongoing loss of skeletal muscle and fat mass that cannot be fully reversed by conventional nutritional support [1]. Cancer cachexia occurs in approximately 80% of cancer patients, and is the primary cause of death in 22–30% of all cancer patients [2, 3]. Cancer cachexia significantly reduces tolerance to antineoplastic therapy and decreases quality of life [4, 5]. However, cancer patients are not usually diagnosed with cachexia until they have lost more than 5–7% body mass due to lack of effective early detection markers [6]. There is therefore an urgent need to understand the underlying mechanisms of cancer cachexia to inform the development of new diagnostic and therapeutic targets.

Although muscle wasting is the hallmark of cancer cachexia, the underlying catabolic driver of cancer cachexia comprises more than just proteolytic breakdown of contractile muscle proteins. Depletion of adipose tissue also contributes to the devastating impact of cancer cachexia [7]. Loss of adipose tissue has been reported to be associated with reduced quality of life and shorter survival independent of body mass index (BMI) in advanced cancer patients [8, 9]. Increased lipolysis and fat oxidation, decreased lipogenesis, impaired lipid deposition and adipogenesis, as well as browning of white adipose tissue (WAT) may underlie adipose atrophy in cancer cachexia [10].

In general, there are two classical types adipose tissues; WAT, which is responsible for accumulation of intracellular lipid droplets and brown adipose tissue (BAT) that causes energy dissipation as heat [11]. Distinct thermogenic BAT consists of not only the classical brown adipocytes but also a second type of brown adipocytes, so-called “beige cells”, which appears in WAT depots in response to cold or β3-adrenergic stimuli [12, 13]. Beige adipocytes are also characterized by the expression of uncoupling protein 1 (UCP1), and regarded as inducible brown adipocytes within WAT, a phenomenon termed WAT browning [11, 14]. Mounting evidence shows that WAT browning is responsible for a significant increase in total energy expenditure as found in obesity [15]. Therefore, stimulation of WAT browning via different strategies has become an attractive therapeutic approach for obesity-associated metabolic syndrome [16, 17]. In contrast to obesity, cancer cachexia is characterized by WAT loss. Therefore, although WAT browning is considered to be beneficial for obesity, whether WAT browning is beneficial for cancer cachexia has not been fully elucidated. In addition, WAT browning was detected in cachectic mice [18]. However, the incidence of WAT browning in cachectic gastric and colorectal cancer patients has rarely been reported.

Chronic inflammation as mediated by interleukin-6 (IL-6) and tumor necrosis factor alpha (TNF-α) has been widely investigated as an important regulator of fat wasting in cancer cachexia [3, 10]. However, the relationship between inflammatory cytokines and WAT lipolysis and browning in cachectic patients has also rarely been reported. Whether inflammatory cytokines contribute to adipose depletion in cancer cachexia by accelerating WAT lipolysis and browning therefore remains unclear.

In the present study, we detected WAT lipolysis and browning in subcutaneous WAT of cachectic patients with gastric and colorectal cancer. The relationship between inflammatory cytokines and WAT lipolysis and browning were also analyzed in cachectic patients. The effect of IL-6 on WAT lipolysis and browning was analyzed in cachectic mice.

## Methods

### Patients and sample collection

Subcutaneous WATs were collected during surgery from patients with gastric and colorectal cancer in Zhongshan Hospital of Fudan University during January 1, 2014 to December 31, 2016. Diagnoses of malignant disease were confirmed by postoperative pathological examinations. Exclusion criteria were as follows: 1) patient ages <18 years; 2) patients who received chemotherapy or anti-inflammatory treatment before surgery; and 3) patients with acute or chronic renal and liver failure, acute and chronic hepatitis, diabetes, metabolic acidosis, sepsis, acquired immune deficiency syndrome (AIDS), inflammatory bowel disease, autoimmune disorders, chronic heart failure, hyperthyroidism, and chronic obstructive pulmonary disease. Written informed consent was obtained from all patients. The Ethics Committee of Zhongshan Hospital of Fudan University approved study (No.: B2013-106R). The subjects were divided into three groups: non-cachexia (*n* = 50), early-stage cancer cachexia (*n* = 40), and late-stage cancer cachexia (*n* = 28). Patients were considered cachectic based on criteria from an international consensus [1]. In this study, we defined late-stage cachexia as patients with weight loss >10% in the past 6 months.

Subcutaneous WATs were cut into halves, and one piece was immediately frozen in liquid nitrogen and stored at −80 °C until further analysis, while the other half was fixed in 10% formalin and embedded in paraffin. Blood samples of all patients were collected before surgery and immediately centrifuged at 3000 rpm for 15 min at 4 °C. Serum samples were preserved at −80 °C for further analysis. The clinical characteristics of each patient before surgery, including age, sex, and BMI were recorded.

### Experimental cachexia model and treatments

BALB/c male mice (6–8 weeks of age) weighing 16–20 g were purchased from the Shanghai Laboratory Animal Center, Chinese Academy of Sciences. Mice were housed at 22 ± 1 °C with a 12-h light/dark cycle and had free access to water and conventional diet. Mice were acclimatized to the environment for 1 week before start of study. All animal manipulations were carried out according to the guidelines and regulations for the use of experimental animals by the Chinese Academy of Sciences. All efforts were made to minimize animal suffering, and to use only the number of animals necessary to produce reliable scientific data.

Colon 26/clone 20 cells, which have been reported to induce severe cachexia in BALB/c mice by subcutaneous inoculation, were cultured in Roswell Park Memorial Institute (RPMI)-1640 medium supplemented with 5% fetal bovine serum and 1% penicillin-streptomycin at 37 °C in 5% CO_2_. Mice were randomly assigned to three experimental groups: control group, Colon 26 tumor-bearing group, and anti-IL-6 receptor antibody (eBiosciences, CA, USA) treated tumor-bearing group. On study day 0, 1.0 × 10^6^ cells suspended in 100 μl phosphate-buffered saline (PBS) were injected subcutaneously into the right armpit of mice in tumor-bearing and anti-IL-6 receptor antibody-treated group. An equal volume of PBS without tumor cells was injected into the control group. In the anti-IL-6 receptor antibody-treated group, each mouse received intraperitoneal injection of 10 μg anti-IL-6 receptor monoclonal antibody diluted in 200 μl normal saline every 2 days. Control and tumor-bearing groups received 200 μl PBS. On day 16, the mice were sacrificed via cervical dislocation. Subcutaneous inguinal and epididymal WAT, interscapular BAT, and gastrocnemius muscle were collected and weighed. Specimens were cut into halves, processed and stored as was carried out with human WAT samples.

### Immunohistochemistry and morphological analysis

Human and mouse subcutaneous WATs embedded in paraffin were cut into 5 μm sections. UCP1 immunostaining of all tissues were performed as previously described [19]. Briefly, slides were dehydrated in graded alcohols and xylene. Antigen retrieval was performed with 0.01 M citrate buffer at 95 °C for 20 min at pH 6.0. Slides were incubated with diluted primary antibodies (anti-UCP1, 1:100 dilutions) for 12 h. The slides were then incubated with biotinylated secondary antibody for 1 h, peroxidase-labeled streptavidin for 15 min, and diaminobenzidine and hydrogen peroxide chromogen substrate plus diaminobenzidine enhancer for 10 min, followed by counter staining with Mayer’s hematoxylin. Images were obtained with a × 40 objective lens. Adipocyte sizes of WAT were manually traced and quantified using ImageJ software.

### Real time PCR analysis

Total RNA was isolated from human and mouse subcutaneous WAT using TRIzol Reagent (Invitrogen, CA, USA) according to the manufacturer’s recommendations. RNA concentrations were quantified by NanoDrop 2000 spectrophotometer and integrity was determined by gel electrophoresis. Complementary DNA was synthesized from 1 μg total RNA using cDNA Synthesis kit (Takara, Dalian, Japan) following the manufacturer’s protocols. Gene expression analysis was performed using PrimeScript RT master mix (Takara, Dalian, Japan) in StepOnePlus Real-Time system (Applied Biosystems, CA, USA). Relative gene expression levels were calculated using 2^-∆∆Ct^ and compared with 18sRNA as internal control. Primers used are shown in Table 1.

**Table 1:** Primers used for real-time PCR analyses

Genes		Human	Mouse
Atgl	F	TCCTCGGCGTCTACTACGTC	CAACGCCACTCACATCTACG
	R	CTCAATGAACTTGGCACCAG	AGCAGGCAGGGTCTTCAGT
Hsl	F	AACTGCCAGCTGCCTTAAAA	CCTCAAAGTCAAACCCTCCA
	R	TTCCCTCACGGGAGATATTG	GTGCGTAAATCCATGCTGT
Cgi58	F	GTGCCCTAGGATTGGACAAA	TGTGTCCCCTGCACTTACAA
	R	GGCTCTGATCCAAACTGGAA	AAAATTCAGGGCCCAAAGT
Ucp1	F	GTGTGCCCAACTGTGCAATG	CACCTTCCCGCTGGACACT
	R	CCAGGATCCAAGTCGCAAGA	CCCTAGGACACCTTTATACCTAATGG
Tmem26	F	ATGGAGGGACTGGTCTTCCTT	ACCCTGTCATCCCACAGAG
	R	CTTCACCTCGGTCACTCGC	TGTTTGGTGGAGTCCTAAGGTC
Tbx1	F	ACGACAACGGCCACATTATTC	GGCAGGCAGACGAATGTTC
	R	CCTCGGCATATTTCTCGCTATCT	TTGTCATCTACGGGCACAAAG
Eva1	F	GGAATCCTGAGCGGTACGATG	CCACTTCTCCTGAGTTTACAGC
	R	CTGGCAGGTGTATGTCCCATT	GCATTTTAACCGAACATCTGTCC
Pdk4	F	GGAGCATTTCTCGCGCTACA	CCGCTTAGTGAACACTCCTTC
	R	ACAGGCAATTCTTGTCGCAAA	TCTACAAACTCTGACAGGGCTTT

### Serum determinations

Human serum concentrations of albumin, triglyceride, and free fatty acids (FFAs) were determined in the laboratory department of Zhongshan Hospital of Fudan University using commercial enzymatic kits. Serum concentrations of IL-6 and TNF-α were assessed using IL-6 and TNF-α enzyme-linked immunosorbent assay (ELISA) kit according to the manufacturer’s protocols.

### Western blot analysis

Preparation of total protein lysates and western blot analysis were performed as previously described [19]. Primary antibodies (1:1000 dilution) against Cgi58 and Tbx1 (Abcam, UK) were used. Tubulin expression was used as an endogenous control.

### Statistical analyses

Results are expressed as mean ± standard error of the mean (s.e.m.). The mRNA levels of relevant genes were compared between groups with two-tailed Student’s *t* test. The correlations of serum FFA with IL-6 and TNF-α in early- and late-stage cachexia were analyzed using Spearman rank correlation tests. All statistical analyses were performed using GraphPad Prism 5.0. Statistical significance was defined as *P* < 0.05.

## Results

### Clinical characteristics of patients and association between inflammatory cytokines and WAT lipolysis

**Fig. 1:**
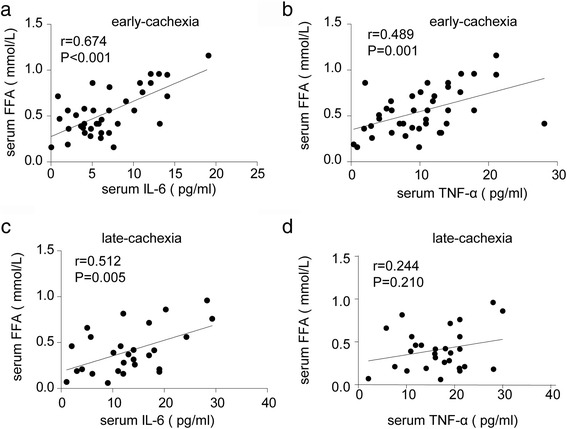
Association between serum inflammatory cytokines and WAT lipolysis in cachectic patients. **a** Correlation analysis between serum IL-6 and FFA in early-stage cachexia (*n* = 40). **b** Correlation analysis between serum TNF-α and FFA in early-stage cachexia (n = 40). **c** Correlation analysis between serum IL-6 and FFA in late-stage cachexia (*n* = 28). **d** Correlation analyses between serum TNF-α and FFA in late-stage cachexia (*n* = 28)

First, we compared the clinical characteristics of the different groups of patients in the study. As shown in Table 2, serum concentration of IL-6 was higher in patients with late-stage cachexia than those without cachexia and early-stage cachexia. Interestingly, serum concentration of TNF-α was increased in only late-stage cachexia. Furthermore, serum albumin and BMI were significantly decreased in late-stage cachectic patients. Although there were no differences in serum triglyceride among the three groups, the concentration of serum FFA was significantly increased in cachectic patients, especially in early-stage cachexia. Correlation analyses showed significantly positive association between serum IL-6 and FFA in both early- and late-stage cachexia (Fig. 1). However, serum TNF-α was positively associated with only serum FFA in early- but not late-stage cachexia (Fig. 1).

**Table 2:** Patient data

	Non-cachexia (*n* = 50)	Early-cachexia (*n* = 40)	Late-cachexia (*n* = 28)
Ages (years)	63.5 ± 11.8	62.1 ± 10.6	59.7 ± 11.3
Female no. (%)	22 (44.4%)	18 (45%)	13 (46.4%)
BMI (kg/m^2^)	23.9 ± 3.5	20.2 ± 3.1^*^	18.3 ± 3.4^*, #^
Serum albumin (g/l)	38.6 ± 4.4	33.7 ± 3.8^*^	30.1 ± 3.1^*, #^
Serum triglyceride (mmol/l)	1.3 ± 0.6	1.6 ± 0.9	1.4 ± 0.6
Serum FFA (mmol/l)	0.32 ± 0.18	0.55 ± 0.25^*^	0.41 ± 0.25 ^#^
Serum IL-6 (pg/ml)	4.6 ± 2.7	7.2 ± 4.3^*^	13.3 ± 7.4^*, #^
Serum TNF-α (pg/ml)	6.8 ± 4.4	10.2 ± 5.0	16.7 ± 6.7^*, #^

^*^P < 0.05 (early stage cachexia VS. non-cachexia; or late stage cachexia VS. non-cachexia;); ^#^*P* < 0.05 (late stage cachexia VS. early stage cachexia)

### WAT lipolysis increased in both early- and late-stage cachexia while WAT browning was detected only in late-stage cachexia

**Fig. 2:**
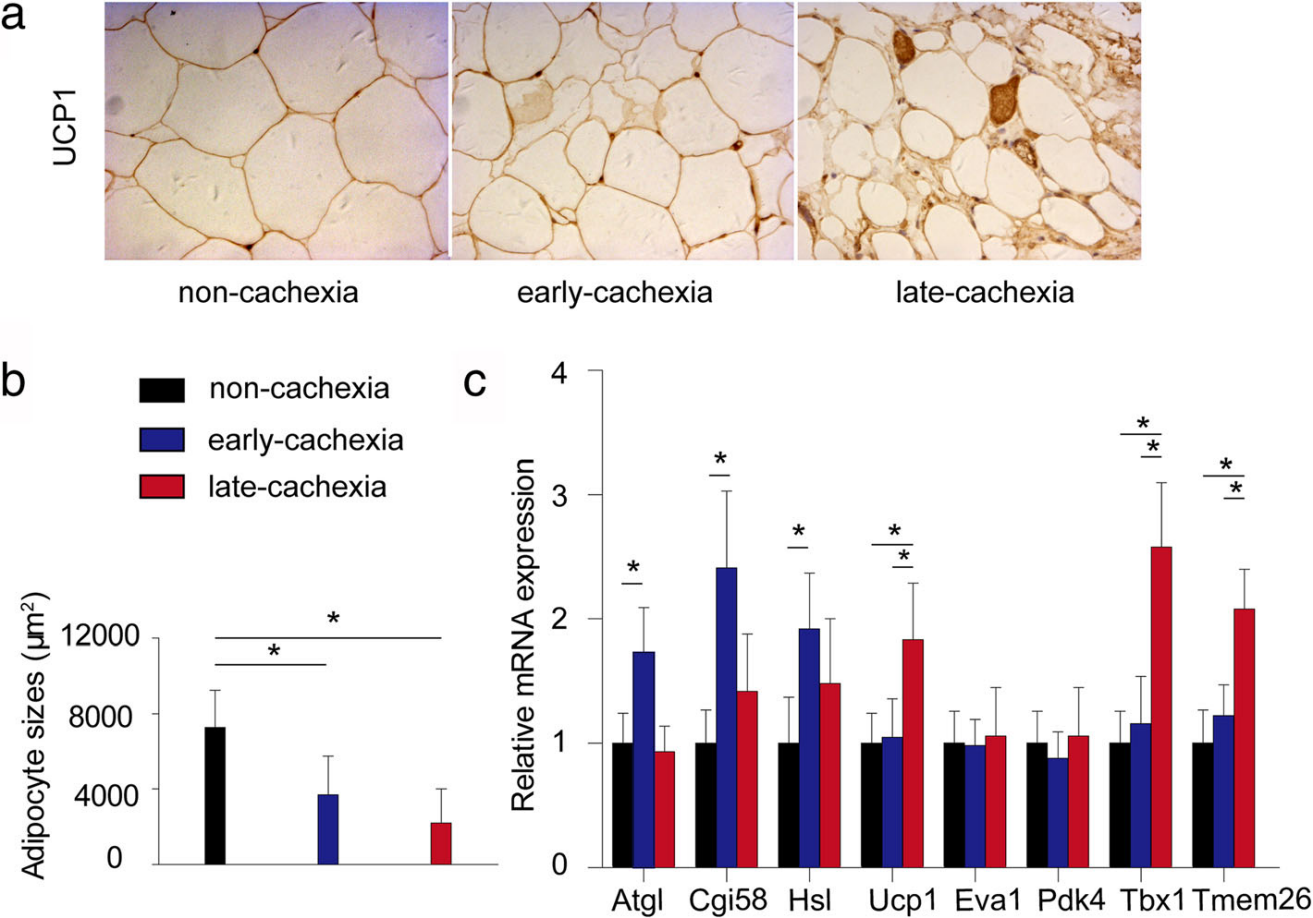
Comparisons of subcutaneous WAT among patients with non-cachexia (*n* = 50), patients with early-stage cachexia (*n* = 40), and patients with late-stage cachexia (*n* = 28). **a** Representative Ucp1 staining of subcutaneous WAT in different groups of patients. **b** Mean sizes of adipocyte in different groups of patients. **c** mRNA expression of WAT lipolysis and browning associated genes in different groups of patients. *P < 0.05

To determine whether human subcutaneous WAT undergoes lipolysis and browning, we investigated WAT morphology and UCP1 expression in subcutaneous WAT of the different groups of patients. There was obvious WAT atrophy in cachectic compared to non-cachectic patients (Fig. 2a). However, no significant difference in adipocyte sizes was detected between early- and late-stage cancer cachexia (Fig. 2b). Interestingly, WAT lipolysis-associated genes (*Atgl, Cgi58*, and *Hsl*) were upregulated in early- but not late-stage cachexia (Fig. 2c).

To determine whether WAT browning occurred in cachectic patients, we found upregulated UCP1 mRNA in patients with late-stage cancer cachexia compared to the other two groups (Fig. 2c). Immunohistochemistry also confirmed the high expression of UCP1 protein in late-stage cancer cachexia (Fig. 2a). We also detected the classical “beige cells”, which were characterized as UCP1-positive and morphologically by multilocular lipid deposits, in 6 of 28 late-stage cachectic patients. To confirm the occurrence of WAT browning, we examined several established brown and beige fat-associated markers in subcutaneous WAT. Interestingly, beige fat-associated genes (*Tmem26* and *Tbx1*) but not brown fat-associated genes (*Eva1* and *Pdk4*) were upregulated in patients with late-stage cachexia (Fig. 2a).

In addition, we found significantly increased serum concentrations of IL-6 and TNF-α in 6 patients with, compared to 22 patients without beige fat cells (data not shown), indicating IL-6 and TNF-α might be associated with WAT browning.

### Anti-IL-6 receptor antibody inhibited WAT lipolysis and browning in cachectic mice

**Fig. 3:**
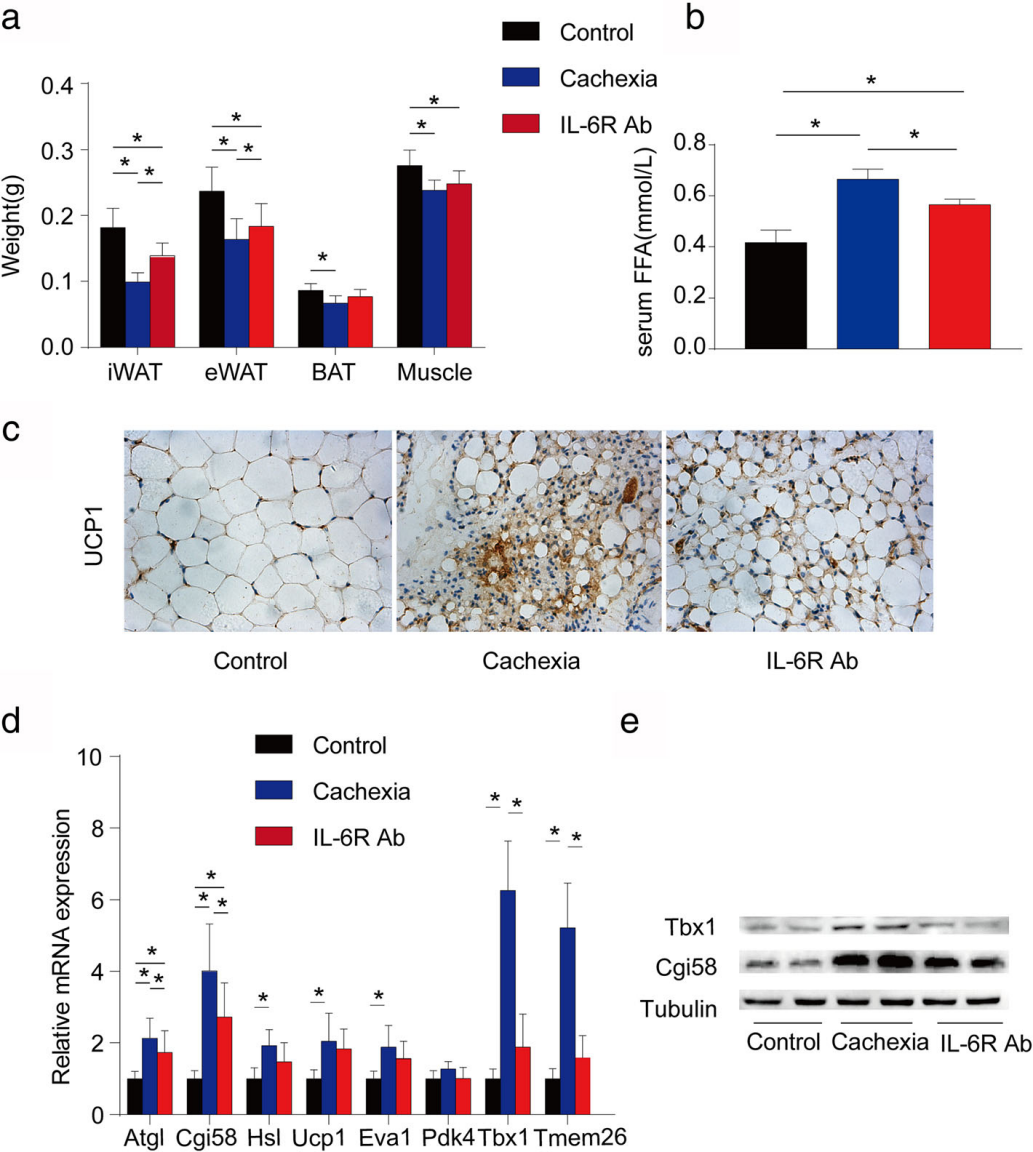
Comparison among control mice (*n* = 8), cachexic mice (*n* = 8), and anti-IL-6 receptor antibody (IL-6R Ab) treated mice (*n* = 8). **a** Weight of inguinal WAT, epididymal WAT, BAT, and skeletal muscle in different groups of mice. **b** Serum concentrations of FFA in different groups of mice. **c** Representative Ucp1 staining of inguinal WAT in different groups of mice. **d** mRNA expression of WAT lipolysis and browning associated genes of inguinal WAT in different groups of mice. **e** The protein expression of Cgi58 and Tbx1 of inguinal WAT in different groups of mice *P < 0.05

To clarify whether IL-6 promotes WAT lipolysis and browning in cancer cachexia, we used anti-IL-6 receptor antibody to inhibit IL-6 in Colon 26 tumor-bearing cachectic mice. Inguinal and epididymal WAT, BAT, and skeletal muscle were significantly decreased in cachectic mice, while they were partly preserved (especially inguinal WAT) in anti-IL-6 receptor antibody-treated mice (Fig. 3a). Serum concentration of FFA was increased in cachectic mice, but partly decreased in anti-IL-6 receptor antibody-treated mice (Fig. 3b). Analysis of inguinal WAT also showed significant cell atrophy and positive UCP1 staining in cachectic mice, and a protective effect by anti-IL-6 receptor antibody (Fig. 3c). Three WAT lipolysis-associated genes (*Atgl, Cgi58*, and *Hsl*) were significantly upregulated in cachectic compared to control mice. However, only *Atgl* and *Cgi58* were significantly upregulated in anti-IL-6 receptor antibody-treated mice (Fig. 3d). Interestingly, two beige fat-associated genes (*Tmem26* and *Tbx1*) and one brown fat-associated gene (*Eva1*) were upregulated in cachectic compared to control mice. However, none of these genes was significantly upregulated in anti-IL-6 receptor antibody-treated mice (Fig. 3d). As shown in Fig. 3e, the protein expression levels of *Cgi58* and *Tbx1* were consist with the mRNA expression.

## Discussion

A key feature of cancer cachexia is loss of WAT because of increased adipocyte lipolysis and other mechanisms. Given that serum FFA mainly originates from lipolysis of WAT, we first examined changes in serum FFA in cachectic patients. Interestingly, serum FFA levels were higher in early- than late-stage cachexia, indicating that WAT lipolysis might be the dominant mechanism in early-stage cancer cachexia.

In spite of increased WAT lipolysis, WAT browning has been proposed as another contributor to WAT atrophy during cancer cachexia [10]. In this study, we report for the first time the occurrence of WAT browning in late- but not early-stage cancer cachexia in humans. The results suggest WAT browning might be a terminal phenomenon that accelerates WAT atrophy in late-stage cancer cachexia.

It is well established that there is a link between cachexia and systemic inflammation. However, the mechanisms and effects of this inflammatory response are not clear. Pro-inflammatory cytokines (e.g., TNF-α and IL-6) produced by tumor or host tissue due to tumor presence leads to both systemic and local inflammation in cancer [20, 21]. However, data on local adipose tissue inflammation in cancer are inconsistent, being reported as either increased or unchanged [22, 23]. There are also conflicting results on whether levels of TNF-α are increased in cancer patients with weight loss [3]. A trial of anti-TNF-α antibodies in patients with cancer cachexia has also shown no benefit [24]. In our study, serum TNF-α increased with decreasing body weight. However, serum TNF-α was positively associated with serum FFA in early- but not late-stage cancer cachexia, which suggests TNF-α may accelerate WAT lipolysis in early-stage cancer cachexia, with reduced effect in late-stage cachexia.

In contrast to TNF-α, circulating levels of IL-6 have been shown to correlate with weight loss in cancer patients, and importantly, IL-6 levels correlated with reduced survival [3]. We also found positive association between serum IL-6 and FFA in both early- and late-stage cachexia, suggesting IL-6 might induce weight loss in cancer cachexia by accelerating WAT lipolysis. However, trials of a monoclonal anti-IL-6 antibody in weight-losing lung cancer patients showed no significant effect on loss of lean body mass [25]. Therefore, whether the effect of IL-6-induced weight loss in cancer cachexia is mainly through WAT but not muscle loss need further study.

Several studies have reported the occurrence of WAT browning in mouse models of cancer cachexia [18, 26]. However, whether WAT browning occurs in cachectic gastric and colorectal cancer patients remains unclear. In this study, we found classical beige cells in only 6 patients with late-stage cachexia. To the best of our knowledge, this is the first report on the presence of WAT browning in a large sample of gastric and colorectal cancer patients. Our results indicate WAT browning might not be a common phenomenon in patients with cancer cachexia. We also found serum concentrations of IL-6 and TNF-α to be significantly increased in 6 patients with compared to 22 patients without beige fat cells, indicating IL-6 and TNF-α might accelerate WAT browning.

Fat loss observed in cachectic patients is thought to occur via breakdown of adipose tissue (mainly WAT) [13]. In cancer cachexia, lipolysis and lipid wasting may occur to an extent before muscle loss [27]. Consistent with the findings in a mouse model of colon cancer that demonstrated an increase in protein kinase-A-mediated lipolysis in early-stage cachexia [28], we also found upregulated WAT lipolysis-associated genes in early-stage cancer cachexia. WAT lipolysis-associated genes were not upregulated in late-stage cachexia, which may explain the lower serum FFA levels in late- compared to early-stage cachexia.

Recent data suggest there are two distinct types of brown fat: classical BAT derived from myogenic factor 5 (myf-5) lineage cells and UCP1-positive cells referred to as beige adipocytes that emerge in white fat from a non-myf-5 lineage [29, 30]. BAT was once thought to be present in only rodents and neonates [31]. However, highly metabolically active BAT was identified in adult humans via positron emission tomographic and computed tomographic (PET/CT) scanning [32, 33]. Recent data suggested that human BAT consist of mainly beige adipocytes, indicating WAT browning occurs in adult humans [11, 34]. In our study, markers of beige adipocytes characterized the UCP1-positive cells detected in late-stage cachectic patients. Although both beige and brown fat-associated genes were upregulated in cachectic mice, the markers of beige adipocytes were more significantly elevated than those of brown adipocytes. Taken together, the UCP1-positive cells detected in WAT in both cachectic cancer patients and mice were beige adipocytes.

In cancer cachectic mice, we found inhibition of IL-6 significantly preserved the weights of inguinal and epididymal WAT. However, weights of BAT and muscle were not significantly preserved by inhibition of IL-6. These findings in combination with the inhibition of WAT lipolysis and browning-associated genes in inguinal WAT of anti-IL-6 receptor antibody-treated mice suggest IL-6 might induce inguinal WAT atrophy by accelerating WAT lipolysis and browning. However, whether this proposed mechanism occurs in humans need further investigation.

## Conclusion

Our results provide direct confirmatory evidence for the occurrence of WAT browning in cachectic gastric and colorectal cancer patients. We also suggest that IL-6 might induce WAT atrophy during cancer cachexia by accelerating WAT lipolysis and browning. These data suggest inhibition of IL-6 might be a promising approach to ameliorate fat loss in cancer cachexia, at least for gastric and colorectal cancer patients.

## Abbreviations

Atgl: Adipose triglyceride lipase
BAT: Brown adipose tissue (BAT)
BMI: Body mass index
Cgi58: Comparative gene identification-58
Eva1: Epithelial V-like antigen 1
FFA: Free fatty acid
Hsl: Hormone-sensitive lipase
IL-6: Interleukin-6
PBS: Phosphate-buffered saline
Pdk4: Pyruvate dehydrogenase kinase 4
Tbx1: T-box transcription factor
Tmem26: Transmembrane protein 26
TNF-α: Tumor necrosis factor alpha
UCP1: Uncoupling protein 1
WAT: White adipose tissue

## References

[1] Fearon K, Strasser F, Anker SD, Bosaeus I, Bruera E, Fainsinger RL (2011). Definition and classification of cancer cachexia: an international consensus. Lancet Oncol.

[2] Fearon K, Arends J, Baracos V (2013). Understanding the mechanisms and treatment options in cancer cachexia. Nat Rev Clin Oncol.

[3] Fearon KC, Glass DJ, Guttridge DC (2012). Cancer cachexia: mediators, signaling, and metabolic pathways. Cell Metab.

[4] Fearon KC, Voss AC, Hustead DS, Cancer Cachexia Study G (2006). Definition of cancer cachexia: effect of weight loss, reduced food intake, and systemic inflammation on functional status and prognosis. Am J Clin Nutr.

[5] Bachmann J, Buchler MW, Friess H, Martignoni ME (2013). Cachexia in patients with chronic pancreatitis and pancreatic cancer: impact on survival and outcome. Nutr Cancer.

[6] Gallagher IJ, Stephens NA, MacDonald AJ, Skipworth RJ, Husi H, Greig CA (2012). Suppression of skeletal muscle turnover in cancer cachexia: evidence from the transcriptome in sequential human muscle biopsies. Clin Cancer Res.

[7] Tsoli M, Swarbrick MM, Robertson GR (2016). Lipolytic and thermogenic depletion of adipose tissue in cancer cachexia. Semin Cell Dev Biol.

[8] Fouladiun M, Korner U, Bosaeus I, Daneryd P, Hyltander A, Lundholm KG (2005). Body composition and time course changes in regional distribution of fat and lean tissue in unselected cancer patients on palliative care--correlations with food intake, metabolism, exercise capacity, and hormones. Cancer.

[9] Murphy RA, Wilke MS, Perrine M, Pawlowicz M, Mourtzakis M, Lieffers JR (2010). Loss of adipose tissue and plasma phospholipids: relationship to survival in advanced cancer patients. Clin Nutr.

[10] Ebadi M, Mazurak VC (2014). Evidence and mechanisms of fat depletion in cancer. Nutrients.

[11] Wu J, Bostrom P, Sparks LM, Ye L, Choi JH, Giang AH (2012). Beige adipocytes are a distinct type of thermogenic fat cell in mouse and human. Cell.

[12] Barbatelli G, Murano I, Madsen L, Hao Q, Jimenez M, Kristiansen K (2010). The emergence of cold-induced brown adipocytes in mouse white fat depots is determined predominantly by white to brown adipocyte transdifferentiation. Am J Physiol Endocrinol Metab.

[13] Vaitkus JA, Celi FS (2017). The role of adipose tissue in cancer-associated cachexia. Exp Biol Med (Maywood).

[14] Guerra C, Koza RA, Yamashita H, Walsh K, Kozak LP (1998). Emergence of brown adipocytes in white fat in mice is under genetic control. Effects on body weight and adiposity. J Clin Invest.

[15] Harms M, Seale P (2013). Brown and beige fat: development, function and therapeutic potential. Nat Med.

[16] Yoneshiro T, Aita S, Matsushita M, Kayahara T, Kameya T, Kawai Y (2013). Recruited brown adipose tissue as an antiobesity agent in humans. J Clin Invest.

[17] Yoneshiro T, Saito M (2013). Transient receptor potential activated brown fat thermogenesis as a target of food ingredients for obesity management. Curr Opin Clin Nutr Metab Care.

[18] Petruzzelli M, Schweiger M, Schreiber R, Campos-Olivas R, Tsoli M, Allen J (2014). A switch from white to brown fat increases energy expenditure in cancer-associated cachexia. Cell Metab.

[19] Han J, Meng Q, Xi Q, Zhang Y, Zhuang Q, Han Y (2016). Interleukin-6 stimulates aerobic glycolysis by regulating PFKFB3 at early stage of colorectal cancer. Int J Oncol.

[20] Lin WW, Karin M (2007). A cytokine-mediated link between innate immunity, inflammation, and cancer. J Clin Invest.

[21] Bing C, Trayhurn P (2009). New insights into adipose tissue atrophy in cancer cachexia. Proc Nutr Soc.

[22] Batista ML, Olivan M, Alcantara PS, Sandoval R, Peres SB, Neves RX (2013). Adipose tissue-derived factors as potential biomarkers in cachectic cancer patients. Cytokine.

[23] Dahlman I, Mejhert N, Linder K, Agustsson T, Mutch DM, Kulyte A (2010). Adipose tissue pathways involved in weight loss of cancer cachexia. Br J Cancer.

[24] Jatoi A, Ritter HL, Dueck A, Nguyen PL, Nikcevich DA, Luyun RF (2010). A placebo-controlled, double-blind trial of infliximab for cancer-associated weight loss in elderly and/or poor performance non-small cell lung cancer patients (N01C9). Lung Cancer.

[25] Bayliss TJ, Smith JT, Schuster M, Dragnev KH, Rigas JR (2011). A humanized anti-IL-6 antibody (ALD518) in non-small cell lung cancer. Expert Opin Biol Ther.

[26] Seale P, Conroe HM, Estall J, Kajimura S, Frontini A, Ishibashi J (2011). Prdm16 determines the thermogenic program of subcutaneous white adipose tissue in mice. J Clin Invest.

[27] Das SK, Eder S, Schauer S, Diwoky C, Temmel H, Guertl B (2011). Adipose triglyceride lipase contributes to cancer-associated cachexia. Science.

[28] Kliewer KL, Ke JY, Tian M, Cole RM, Andridge RR, Belury MA (2015). Adipose tissue lipolysis and energy metabolism in early cancer cachexia in mice. Cancer Biol Ther.

[29] Wu J, Cohen P, Spiegelman BM (2013). Adaptive thermogenesis in adipocytes: is beige the new brown?. Genes Dev.

[30] Peirce V, Carobbio S, Vidal-Puig A (2014). The different shades of fat. Nature.

[31] Aherne W, Hull D (1966). Brown adipose tissue and heat production in the newborn infant. J Pathol Bacteriol.

[32] Cypess AM, Lehman S, Williams G, Tal I, Rodman D, Goldfine AB (2009). Identification and importance of brown adipose tissue in adult humans. N Engl J Med.

[33] Saito M, Okamatsu-Ogura Y, Matsushita M, Watanabe K, Yoneshiro T, Nio-Kobayashi J (2009). High incidence of metabolically active brown adipose tissue in healthy adult humans: effects of cold exposure and adiposity. Diabetes.

[34] Cannon B, Nedergaard J (2012). Cell biology: neither brown nor white. Nature.

